# A Case Report of Fructosamine’s Unreliability as a Glycemic Control Assessment Tool in Nephrotic Syndrome

**DOI:** 10.7759/cureus.1694

**Published:** 2017-09-16

**Authors:** Sri HarshaVardhan Senapathi, Ravi Bhavsar, Regina Kaur, Paul Kim, Issac Sachmechi

**Affiliations:** 1 Internal Medicine, Mount Sinai Icahn School of Medicine Queens Hospital New York

**Keywords:** diabetes, fructosamine, glycemic markers, nephrotic syndrome, hba1c

## Abstract

Fructosamine (FA) is a glycated primary amine widely used as an alternative method for the assessment of glycemic control when glycosylated hemoglobin (HbA1c) measurement is unreliable or if there is a need for short-term glycemic control monitoring. We report a case of a 36-year-old male patient with a six-year history of poorly controlled type 2 diabetes mellitus and nephrotic syndrome. As the nephrotic syndrome progressed, we observed a decline in his serum FA levels that did not correlate with his increased HbA1c due to significant albuminuria. This case report highlights the unreliability of FA in patients with nephrotic syndrome and the significance of other glycemic markers.

## Introduction

The term fructosamine (FA) typically refers to all ketoamine linkages that result from the glycation of serum proteins. Glycation is a non-enzymatic process, also known as the Maillard reaction, in which glucose and other sugars react spontaneously with free amino terminal residues of serum proteins [1]. The elevated serum glucose concentration increases the glycation of serum proteins, which in turn leads to a rise in serum FA. Hence, an elevated serum glucose directly correlates with increased levels of serum FA. The short half-life of serum proteins (14 to 21 days) makes FA a valuable substitute for glycosylated hemoglobin (HbA1c) for short-term glucose monitoring [1]. FA is a good alternative to HbA1c for patients with chronic kidney disease, hemoglobinopathies, and gestational diabetes mellitus. FA measures glycated serum proteins, which makes it potentially unreliable as a measure of glycemia for patients with increased protein turnover [1-2].

## Case presentation

Our patient was a 36-year-old obese man with type 2 diabetes mellitus (T2DM) treated with metformin (1000 mg, twice daily), pioglitazone (45 mg, once daily), and 70/30 insulin (twice daily, consisting of a 40-unit dose and a 44-unit dose). Despite numerous modifications to his medications, his HbA1c levels remained elevated. Initially, there were significant discrepancies between his elevated HbA1c levels and home glucose monitoring record, which prompted us to use FA along with HbA1c as a quantitative measure of glycemic control. The patient’s HbA1c and FA were in correlation until he developed nephrotic syndrome. As his proteinuria worsened (3.6 g/24 hours), the correlation between FA (0.330 mmol/L) and HbA1c (13.3%) was lost despite borderline-low albumin levels of 32 g/L. His FA levels dropped further as the proteinuria worsened. Table 1 shows the complete progression of the patient’s glycemic parameters during his illness.

**Table 1:** Progression of patient’s glycemic parameters Abbreviations: HbA1c, glycosylated hemoglobin; FA, fructosamine; GFR, glomerular filtration rate; NP, not performed.

Parameter	Date						
	12/01	6/02	6/03	4/04	12/05	8/06	3/07
Serum Glucose (mg/dL)	183	124	256	78	278	115	123
HbA1c (%)	14	11.1	9.7	8.4	13.3	9.9	9.6
FA (mmol/L)	NP	0.454	NP	NP	0.330	0.221	0.206
Predicted FA (mmol/L)	NP	0.558	NP	NP	0.680	NP	NP
Albumin (g/L)	35	37	29	35	32	27	27
24-Hour Urine Protein (g/24 hr)	0.3	NP	1.0	1.2	3.6	4.9	NP
Spot Urine Microalbumin (mg/dL)	NP	34	307	1070	NP	NP	NP
Creatinine (mg/dL)	0.9	0.8	1.2	1.3	1.5	1.6	2.1
GFR (mL/min)	105	120	75	68	57	53	38

## Discussion

Fasting plasma glucose and HbA1c are conventional methods for the measurement of glycemic control in patients with T2DM. Despite HbA1c being the most accurate marker for the assessment of glycemic control, its reliability is reduced in the presence of conditions that compromise red blood cell function (e.g., hemoglobinopathies, anemias, and renal disease) due to the low levels of hemoglobin resulting in falsely elevated HbA1c values. FA, on the other hand, is independent of hemoglobin, which makes it an effective alternative to HbA1c [1].

FA typically measures glycated albumin (80% of FA), glycated lipoproteins, and globulins. The increased plasma glucose concentration characteristic of T2DM causes an increase in the glycation of serum proteins resulting in elevated FA levels [1]. However, due to the effect of serum proteins on FA, there are some limitations in using this test in patients with hypoalbuminemia, hyperglobulinemia, and paraproteinemia [2]. There have been numerous studies that discuss the efficacy of FA measurement in conditions with altered protein turnover, and some studies suggest using correction formulae for an accurate measurement of FA in cases with deranged serum protein levels [3-4].

Baker et al. suggested that there was no linear relation between FA and serum albumin or total protein concentrations if the serum albumin levels remained above 30 g/L; above this threshold, FA remains a reliable tool of glycemic measurement [5]. However, our case demonstrated a drop in FA levels, even with albumin levels higher than 30 g/L (as shown in Table 1). From this point, the FA levels lost their correlation with the HbA1c levels. Van Dieijen et al. studied FA values in patients with albumin levels both above and below 30 g/L. This study concluded that the influence of albumin concentration on FA is present over the whole range rather than concentrations less than 30 g/L, as suggested by Baker [3,5]. We observed similar findings and strongly believe that the drop in FA levels was most likely secondary to the increased protein turnover caused by nephrotic syndrome. Additionally, Constanti et al. studied the use of FA in subjects with and without nephrotic syndrome having similar glycemic values and concluded that FA was not useful to assess glycemic control in patients with nephrotic syndrome irrespective of serum albumin levels [2].

Van-Dieijen and Howey proposed correction formulae for FA values in patients with altered albumin concentrations, to increase the reliability of the FA assay [3-4]. Cohen et al. proposed a formula to predict FA from HbA1c. These formulae are listed in Table 2 [3,4,6].

**Table 2:** Correction of fructosamine Abbreviations: FAc, corrected fructosamine.

Study	Corrected Fructosamine Values	Corrected FA (in our case) on 12/05
Van-Dieijen et al. [3]	FAc (mmol/L) = FA – 0.0023 x (serum albumin in g/L)	0.256 mmol/L
Howey et al. [4]	FAc (mmol/L) = FA + 0.03 (40 – serum albumin)	0.570 mmol/L
Cohen et al. [6]	Fructosamine (µmol/L) = (HbA1c – 1.61) X 58.82	0.680 mmol/

Applying the above formulae to our case, we observed that the corrected FA values were not consistent with one another. Overall, FA is not a reliable marker for glycemic control in patients with T2DM and nephrotic syndrome even when the necessary corrections are applied. The mechanisms of the other available glycemic markers are discussed below.

Glycated albumin (GA) is another glycemic marker that has shown good promise as an alternative to HbA1c in patients with advanced stages of chronic kidney disease (CKD) undergoing hemodialysis. GA is considered superior to FA as it measures the ratio of glycated albumin to total albumin and not just the amount of glycated albumin in serum [1]. Also, unlike FA, GA values are not affected by other serum proteins like globulins and lipoproteins. However, studies suggest that GA should not be used in situations where FA is unreliable as both these markers depend on albumin levels and will be affected in patients with nephrotic range proteinuria [7].

A recently introduced marker for assessing glycemia is 1,5 anhydroglucitol (1,5 AG). A naturally occurring dietary polyol, 1,5 AG is filtered and completely reabsorbed by the kidneys in euglycemic states, leading to stable serum concentrations [8]. However, 1,5 AG’s absorption is competitively inhibited by glucose in hyperglycemic states, resulting in lower serum values in patients with T2DM, enabling it to assess glycemic control over the past 24 hours to 14 days. Although it appears to be a good estimate of postprandial glucose surge, limited data suggest its superiority over its predecessors [9]. It has been used effectively in CKD stages 1 and 2 but tends to show falsely high values in advanced stages due to poor glomerular filtration rates. Currently, there has been little information suggesting its use in patients with altered serum proteins [8].

Lastly, continuous glucose monitoring remains the most accurate method to assess glycemic states in patients with CKD and nephrotic range proteinuria, as it is not influenced by altered protein or hemoglobin levels.

We have listed the sources of errors for all the glycemic markers in Table 3 [8].

**Table 3:** Sources of error Abbreviations: A1c, glycated hemoglobin; 1,5-AG, 1,5 anhydrogluticol; CKD, chronic kidney disease; RBC, red blood cell.

	A1C	Fructosamine	Glycated Albumin	1,5-AG
Mechanism	Conditions or treatments that alter RBC half-life	Conditions or treatments that alter protein metabolism	Conditions or treatments that alter protein metabolism	Conditions or treatments that alter renal function or threshold for glucose
Falsely high values	Iron deficiency Anemia hemoglobinopathies Race: African, American, Hispanic, Asian	Hypothyroidism Hypogammaglobinemia Paraproteinemia Hyperalbumenic states	Hypothyroidism Hyperalbunemic states	CKD stages 4-5
Falsely low values	Hemolysis Reticulocytosis Hemoglobinopathies Post-hemorrhage or post-transfusion Drugs: iron, erythropoietin, dapsone Uremia Splenomegaly Dapsone Vitamin C and E	Hypoalbuminemia: protein-losing enteropathy, nephrotic syndrome, liver failure Hyperthyroidism Hyperuricemia Hypertriglyceridemia Nonalcoholic fatty liver disease	Hypoalbuminemia: protein-losing enteropathy, nephrotic syndrome, liver failure Hyperthyroidism Nonalcoholic fatty liver disease Cirrhosis of the liver	Pregnancy Chronic liver disease Glucokinase–maturity onset diabetes of the young

## Conclusions

In cases where HbA1c is not reliable, the clinicians will usually proceed to using FA to assess glycemic control as the next-best option. While FA can be a very useful indicator of glycemic control, it still has several limitations in conditions associated with altered protein levels. This case aims to raise awareness of FA being an unreliable marker of glycemic control in patients with nephrotic syndrome irrespective of their serum albumin or total protein levels.
